# A new approach to APCs

**DOI:** 10.1038/s44319-024-00138-1

**Published:** 2024-04-29

**Authors:** Fiona M Watt

**Affiliations:** Directors’ Unit, EMBL Heidelberg, Meyerhofstr. 1, 69117 Heidelberg, Germany

**Keywords:** Science Policy & Publishing

## Abstract

The article processing charges associated with open-access publishing are unaffordable for some scientists. One solution is to break the APC down into its component parts, enabling scientists to spread the costs over multiple providers and several years.

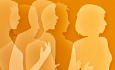

The concept of open-access (OA) sounds great—who would disagree with the idea that research publications should be freely available for everyone to read? But publishing costs money, and that money has to come from somewhere. In the past, publishers obtained their revenue by charging a subscription fee, which was usually paid by institutional libraries. Now OA journals usually require an article processing charge (APC) for publishing accepted manuscripts. The irony is that the old inequality—scientists were excluded from reading papers because their libraries did not subscribe to the journal—has been replaced by a new inequality: scientists are excluded from publishing their work because they cannot afford the APC.

Discussion about APCs tends to treat them as a fixed unit of cost—typically several thousand dollars—and frame the question of who pays: is it the author or the library? Ultimately, whoever is funding the research pays the APC, whether by a block grant to the researcher’s host institution or by expecting the scientist to pay from his or her own research grant. While the former may appear preferable from the scientist’s perspective, the money for a block grant still has to come from somewhere and will typically involve top-slicing the funder’s existing research budget. Yet, I think there is a new way to pay—and that is by breaking the APC down into its component parts.

## Viewing APCs from multiple perspectives

During my career as a cell biologist, I have been involved in both science publishing and science funding. Before OA became mainstream, I was editor-in-chief of the not-for-profit *Journal of Cell Science*, and income from the journal was, and still is, used to support scientists in a range of ways, including grants for conferences and exchange visits. More recently, I was a Deputy Editor at *eLife*, an OA journal that receives funding from the Wellcome Trust, Howard Hughes Medical Institute (HHMI) and Max Planck Society. I stepped down from *eLife* to become Executive Chair of the Medical Research Council, part of the UK government funding agency UK Research and Innovation (UKRI), which provides grants to individual researchers and their host organisations.

Now, as EMBO Director, my dual interests in science publishing and science funding have coalesced. EMBO funds research in a variety of ways including postdoctoral fellowships, conference and travel grants and start-up grants for early-career PIs. We also run EMBO Press, which encompasses multiple journals and the journal-agnostic peer-review platform *Review Commons*. EMBO receives funding from more than 30 government agencies, ranging from countries such as Switzerland and Germany that invest a high proportion of their GDP in research, to countries such as Malta and Croatia, with much smaller research budgets. Unsurprisingly, the different EMBO-associated countries have different approaches to funding OA. To help scientists without ready access to APC funding, we are currently able to publish a small number of EMBO Press papers at no cost to corresponding authors based in EMBO partner countries who meet certain eligibility criteria. We can also apply a waiver scheme where necessary to ensure no author is excluded from publishing by their inability to pay the charges.

## Breaking down APCs into their component parts

At the moment the APC is paid to the journal where the paper is published—it does not cover costs incurred along the way, such as multiple rounds of editorial and peer review at other journals that went on to reject the paper. Moreover, the more papers are published, the more APCs are received by the journal, effectively decreasing the cost of rejection. Major publishers now own hundreds or even thousands of journals, subsidising the costs of their highly selective journals with the APCs from high-volume, high-acceptance rate journals.

How could an APC be split into its component parts? We can break down the publishing process into three main steps: peer review; pre-publication quality checks; and preparing the accepted manuscript for publication; and dissemination and archiving. Some publishers, including EMBO Press, report on their income and expenses. In 2023, 55% of the cost of publishing an EMBO Press paper comprised office costs—primarily editors’ salaries and administration— while 40% was incurred by outsourced publishing services and digital platforms. Thus, roughly 60% of the actual cost of publishing a paper is editorial, peer review and quality checking.

## Peer review

Journal-agnostic peer review was pioneered over 10 years ago by initiatives such as Rubriq (Van Noorden, 2013) and has evolved rapidly since. In the *Review Commons* model, authors receive the reviews of their manuscript, craft a response and then have the option to submit the entire package for consideration by one of almost 30 partner journals in addition to posting ‘refereed preprints’ onto the preprint platform *BioRxiv*. Sharing peer reviews saves time and money. At present, *Review Commons* is supported by EMBO and HHMI but intends to become financially self-sustaining through a combination of in-kind contributions by editors of some journals and an annual subscription fee for other journals. By avoiding multiple rounds of peer review with new reviewers for each journal, the cost of this component of the APC is not only reduced but can be harmonised across multiple journals.

The proposal that peer review can be funded separately from the total APC is already a reality thanks to a recent EU announcement. It is also consistent with the *eLife* publishing model launched in 2023, in which authors pay a relatively modest fee of US$2000 in return for peer review and decide themselves when their paper should become the version of record. *eLife* is a partner of *Review Commons*.

Incentivising peer review by paying reviewers has been around in various formats for many years, but in practice it is cumbersome to administer and a sustainably priced fee is generally too modest to be effective. An alternative would be to award reviewers tokens/credits for their services that could be redeemed to offset the APCs on their own publications. This approach might help overcome the problem that journals have in finding reviewers, but it would only work if there was a common ‘currency’ across multiple journals. In practice, it would be straightforward to apply, given the large number of journals that are typically owned by one publisher or that have come together under the *Review Commons* and *Life Science Alliance* umbrella.

## Quality checks

What about the other components of the APC? It is in everyone’s interest to spot errors in papers before they are published (Watt, 2023). Tools that help prevent mistakes entering the scientific literature are available both to individual scientists and to journals. Many journals and universities use plagiarism detection software such as TurnitIn, and Grammarly is available as a free plug-in with Microsoft Word. Image duplication detection software such as Proofig is used by some journals. Statistics are already checked in-house in some journals and of course there is statistics software available to individuals (for example, https://www.jmp.com/). We can add into the mix public repositories hosted by major institutions such as NCBI and EMBL-EBI (for example, https://www.ncbi.nlm.nih.gov/geo/) where authors upload their data at the same time as submitting their manuscript to a journal. The types of check will depend to some extent on the types of data being published.

I would suggest that authors could have the option to arrange for these checks themselves prior to submission or pay a journal or other provider to carry them out. An extension of this is that peer review can be broken down into different stages (Amaral, 2022) using different reviewers for different aspects of a paper. Authors could then have the option of a technical/statistical check on top of the conventional quality/novelty/conceptual check.

How would quality checks work in practice? Some sort of certification would be required—a GEO submission number is one example—and journals could specify which types of checks and which providers are necessary and acceptable. Here is an opening for a commercial partnership that is not usually linked to science publishing—one involving pharmaceutical companies. For example, Scolary, a free compendium of software tools, including Rubriq, is funded by Merck KGaA, Darmstadt, Germany. With hindsight, it is obvious that a company that sells pharmaceuticals would have a strong interest in ensuring widespread dissemination of high-quality, reproducible data, given the number of medically-relevant papers that are retracted, often many years after publication, having mislead numerous investigators.

## Dissemination and archiving

Now we come to the final component of the APC: the cost of publication, sharing and archiving. Commercial publishers are often cast as villains because they make a profit out of APCs. But we need to be realistic: commercial publishers are answerable to their investors and shareholders in the same way as any company, while not-for-profit publishers use the surplus on their publishing activities to support scientists in a variety of ways, including fellowships and conferences. Some companies have diversified beyond publishing into other areas, such as data management software for universities (for example, https://www.elsevier.com/solutions/pure/pure-in-action), which collects a range of information about individual researchers, including grants and publications, for internal and external reporting and performance review. In this context, the third aspect of the APC could be provided pro bono by companies to the institutions that use their data management software. Of course, this would risk restoring the old inequalities that were linked to journal library subscriptions. Alternatively, science funders could pay for this element, which is essential to ensure the long-term accessibility of the research they have funded.

## Conclusions

Having seen the enormous effort expended by just one government agency (UKRI) in developing an Open Access strategy, I fear that the main barrier to reforming the APC might be the years that funders have already spent reaching their current positions. It would also require a complete re-think of the publishers’ business model. Nevertheless, as I have shown, some of those changes are already in place, and I think abolishing the lump-sum APC would be a new opportunity for publishers and funders alike.

Would it be too complex to administer? Scientists are used to paying for materials and services from a variety of different suppliers, and the sums involved in paying separately for peer review, quality checks and archiving would each be significantly lower than the single APC. Even if the total cost of publishing a paper is the same as the current APC, by breaking the APC into its component parts, scientists would spread payments across different suppliers and over a longer time frame. Funders, scientists, big Pharma and publishers could consult a menu, select which items are required, and then split the bill.

The end of block APCs will be an about-turn for publishers and funders but will ultimately serve science better. Pilot projects such as *Review Commons* have already established the principle that different aspects of publishing can be deconstructed. Ultimately this new approach will put power into the hands of the scientists and is a step towards greater equity.

### Supplementary information

Peer Review File
