# Supplementary material for: A new approach to APCs
Source: EMBO Rep. 2024 Apr 29;25(5):2149–51. doi: 10.1038/s44319-024-00138-1 (PMC11094111; doi:10.1038/s44319-024-00138-1)
Supplement: Supplementary file 1 — Peer Review File [file 44319_2024_138_MOESM1_ESM.pdf]

EMBO reports

A new approach to APCs

Fiona M. Watt

Corresponding author(s): Fiona M. Watt (fiona.watt@embo.org)

**Review Timeline:**	Submission Date:	10th Mar 24
	Accepted:	8th Apr 24

Editor: Holger Breithaupt

Transaction Report: This article type is not peer reviewed.
